# Hard Boiled: Alcohol Use as a Risk Factor for MDMA-Induced Hyperthermia: a Systematic Review

**DOI:** 10.1007/s12640-021-00416-z

**Published:** 2021-09-23

**Authors:** Jan van Amsterdam, Tibor M. Brunt, Mimi Pierce, Wim van den Brink

**Affiliations:** Department of Psychiatry, Amsterdam University Medical Center, University of Amsterdam, P.O. Box 22660, 1100 DD Amsterdam, The Netherlands

**Keywords:** MDMA, Ecstasy, Alcohol, Interaction, Risk factor, Hyperthermia, Hyponatremia, Fatalities

## Abstract

Although MDMA (ecstasy) is a relatively safe recreational drug and is currently considered for therapeutic use for the treatment of posttraumatic stress disorder (PTSD) and alcohol use disorder (AUD), recreational MDMA use occasionally elicits hyperthermia and hyponatremia, sometimes with a fatal outcome. Specific risk factors for both adverse effects are profuse sweating while vigorously dancing under unfavorable conditions such as high ambient temperatures and insufficient fluid suppletion which result in dehydration. Concomitant use of MDMA and alcohol is highly prevalent, but adds to the existing risk, because alcohol facilitates the emergence of MDMA-induced adverse events, like hyperthermia, dehydration, and hyponatremia. Because of potential health-related consequences of concomitant use of MDMA and alcohol, it is important to identify the mechanisms of the interactions between alcohol and MDMA. This review summarizes the main drivers of MDMA-induced hyperthermia, dehydration, and hyponatremia and the role of concomitant alcohol use. It is shown that alcohol use has a profound negative impact by its interaction with most of these drivers, including poikilothermia, exposure to high ambient temperatures, heavy exercise (vigorous dancing), vasoconstriction, dehydration, and delayed initiation of sweating and diuresis. It is concluded that recreational and clinical MDMA-users should refrain from concomitant drinking of alcoholic beverages to reduce the risk for adverse health incidents when using MDMA.

**Supplementary Information:**

The online version contains supplementary material available at 10.1007/s12640-021-00416-z.

## Introduction

MDMA (ecstasy, 3,4-methylenedioxymethamphetamine) is a worldwide popular recreational drug. Though serious health incidents have been reported, including fatal cases, their incidence is very low especially considering its wide-spread use (van Amsterdam et al. 2020). MDMA is usually consumed in dance clubs and raves, frequently in the context of polysubstance use, i.e., MDMA in combination mainly with alcohol (and cannabis) (Barrett et al. 2005; Breen et al. 2006; Degenhardt et al. 2009; Izco et al. 2007; Mohamed et al. 2011; Schifano 2004; Winstock et al. 2001), probably because alcohol is legal and easily accessible in most countries. Around 90% of all ecstasy users also consume alcohol with over 70% of them at hazardous levels (Morefield et al. 2011; Winstock et al. 2001). In Australia, 65% of regular ecstasy users reported concomitant use of alcohol and MDMA (Breen, et al. 2006) and 35% reported concomitant high-risk alcohol use (Kinner et al. 2012). According to the 2001–2002 national epidemiologic survey in the USA, 95% of MDMA users regularly drink alcohol (Keyes et al. 2008). More recently, MDMA-assisted psychotherapy is considered for the treatment of severe posttraumatic stress disorder (PTSD) (Mitchell et al. 2021) and alcohol use disorder (Sessa et al. 2021), the latter emphasizing the clinical relevance of possible interactions between MDMA and alcohol.

### Non-fatal Incidents

Alcohol was implicated in 75% of all MDMA-related Emergency Department (ED) presentations in 2008–2010 (*n* = 236) in Australia, which was 5.6-fold more than in GHB-related ED-presentations (Horyniak et al. 2014). Between 2009 and 2019, the Dutch Monitor Drug Incidents (MDI) reported 11,729 adverse health incidents related to the use of ecstasy. The proportion of medium to severe intoxications was substantially higher for co-use of MDMA and alcohol (35%) than for MDMA alone (20%) (Schürmann et al. 2020; Vercoulen and Hondebrink 2021). In a case-series of MDMA-related ED-visits, 9 out of 48 patients showed hyperpyrexia (> 37.1 °C) (Williams et al. 1998), and in another sample of 52 ED-patients, 4% showed hyperthermia (defined as body temperature > 41 °C) and 52% of these patients had used MDMA together with alcohol of whom five patients had severe medical complications and one patient a fatal outcome (Liechti et al. 2005). Grunau et al. have compiled 71 cases (of which 25 were fatal) of MDMA-related hyperpyrexia (Grunau et al. 2010; Liechti et al. 2005). Finally, 17 cases of MDMA-related hyponatremia (in 10 cases analytically confirmed) were identified between 1993 and 1996 by the London center of the National Poisons Information Service with two of them fatal (Hartung et al. 2002). More recently, mild MDMA-related hyponatremia was seen in 9 out of 63 recreational MDMA users during an indoor party (van Dijken et al. 2013).

### Fatal Incidents

Of 495 ecstasy-related deaths in England and Wales between 1997 and 2006, 20% involved the use of MDMA alone and 30% MDMA combined with alcohol (Rogers et al. 2009). Alcohol was detected post-mortem in 29–60%, in addition to MDMA, of drug-related fatalities (Forrest 2003; Kaye et al. 2009; Roxburgh and Lappin 2020). However, these figures cannot be converted to a relative risk of co-use of MDMA and alcohol because the absolute prevalence of either MDMA use alone, combined use of MDMA and alcohol, or alcohol use alone cannot be derived from these studies.

### Fatalities Reported in Clinical Context

Only one study (Cohen et al. 2021), covering 1000 reports as part of FDA drug safety surveillance data on the use of MDMA for the treatment of PTSD, presented 62 unique records of the combined use of MDMA and alcohol. This study showed that the risk for a fatal outcome — not per se resulting from hyperthermia — was two-fold higher when MDMA was combined with alcohol compared to the use of MDMA alone (corrected for the use of other substances). However, the incidence such as fatalities was low considering that clinical cases lacking side effects were not reported to the FDA (Food and Drug Administration). Finally, other classes of drugs, like opioids and benzodiazepines, were more often used concomitantly with MDMA than alcohol, but showed (slightly) lower odds ratios for the reported fatalities.

### Hyperthermia and Dehydration

The most frequently seen acute serious adverse effects of recreational MDMA-use are hyperpyrexia (hyperthermia; heatstroke), dehydration, and hyponatremia. In 87 fatal cases related to MDMA and/or related drugs, hyperpyrexia and hyponatremia-related cerebral edema were the two *primary* causes of death in 30 and 9 cases, respectively (Kalant 2001). Deaths due to hyperthermia involved severe dehydration, rhabdomyolysis, multi-organ failure, renal and liver failure, and disseminated intravascular coagulation (Brown and Osterloh 1987; Docherty and Green 2010; Grunau et al. 2010; Hall and Henry 2006; Liechti et al. 2005; Parrott 2012; Rogers et al. 2009). Hospital presentations of severe hyperthermia in ecstasy users have been frequently reported (Hall and Henry 2006; Patel et al. 2005), although the incidence is relatively low considering its wide-spread use. It remains unclear why moderate hyperpyrexia occasionally progresses to severe hyperthermia (Henry et al. 1992). Most reported cases of MDMA-induced hyperthermia appear to be associated with or facilitated by excessive exertion (vigorous dancing) in warm environments associated with insufficient fluid supplementation, but extreme physical activity is not a requirement for MDMA-induced hyperpyrexia (Hall and Henry 2006). If present, the duration and degree of pyrexia are strong indicators for the mortality risk (Grunau et al. 2010; Hall and Henry 2006; Henry 1992; Liechti 2014) implicating that control of hyperthermia is key to survival (Tehan et al. 1993).

Table 1 shows some risk factors of MDMA-induced (fatal) hyperpyrexia. MDMA-related hyperthermia occurred predominantly in hot nightclubs, which suggest that setting is pivotal for this complication (Henry 1992), and stimulant-driven exertional heatstroke plays a role in MDMA-related hyperthermia. Hyperthermia is, however, also observed following MDMA-exposure outside “rave” party settings in the absence of physical activity (Parrott 2012; Patel, et al. 2005). Dehydration (hypohydration) is another risk factor for and a major cause of hyperthermia. Dehydration results from MDMA-induced vasoconstriction, which halts sweats production, conserves blood volume, and thus leads to an increase in core body temperature. Indeed, recreational MDMA users frequently reported “increased body temperature” (90%), “increased sweating” (85%), and “dehydration” (85%) (Davison and Parrott 1997). Others showed that ecstasy users complained about “overheating/sweating” (> 60%) and an “urge to drink/dehydration” (> 80%) during acute MDMA-exposure (Kish et al. 2010) or reported “hot and cold flushes” and “profuse sweating” (39%) (Topp et al. 1999). In an Internet survey of over 200 recreational users, 31% of the respondents felt moderately hot, and 12% reported feeling very or extremely hot, when on MDMA (Parrott et al. 2006). A dry mouth/thirst, reported by 25–88% of recreational MDMA-users (Baylen and Rosenberg 2006), is a predictor of dehydration which predisposes to heat exhaustion, hyperthermia, and heat stroke. Due to MDMA-induced heat production, subjects start shivering and profuse sweating, which is indicative for hyperpyrexia.

**Table 1:** Risk factors of MDMA-induced (fatal) hyperpyrexia

Risk factors	References
Multiple dosing (booster or high doses)	Green et al. (2004); Parrott (2012); Schütte et al. (2013); Topp et al. (1999)
Prolonged and vigorous dancing in hot settings during “rave” dance parties	Dafters (1995); Docherty and Green (2010); Kiyatkin et al. (2014); Parrott et al. (2006); Suy et al. (1999); Winstock et al. (2001)
Reduced fluid intake and dehydration	Bouchama and Knochel (2002); Coris et al. (2004); Montain and Coyle (1992); Sawka (1992); Sawka et al. (1985)
Co-use of MDMA and alcohol	Calle et al. (2019); Calle et al. (2018); Horyniak et al. (2014); Liechti et al. (2005); Palamar et al. (2019); Rogers et al. (2009); Roxburgh and Lappin (2020); Schifano (2004); Schifano et al. (2003)
Hyperthyroidism	Sprague et al. (2018)

As an inhibitor of the secretion of arginine vasopressin (AVP = antidiuretic hormone = ADH) and as a peripheral vasodilator, which increases diuresis and sweating, respectively, alcohol may facilitate dehydration. This may explain why MDMA-related deaths are more likely to occur when alcohol and other substances are co-ingested (Schifano 2004; Schifano et al. 2003).

In addition to dehydration and heatstroke in dance clubs, MDMA may also cause water retention via stimulation of AVP-release. As a result, MDMA may elicit hyponatremia leading to cerebral edema, which can be fatal, as well. As will be outlined in this review, concurrent use of MDMA and alcohol may increase both the risk of hyperthermia and hyponatremia. Although hyperthermia and hyponatremia are two different syndromes with hyponatremia being less prevalent, hyponatremia may arise from hyperthermia (see Fig. 2). In this review, we provide a summary of the available pre-clinical and clinical information to better understand the possible mechanisms leading to hyperpyrexia and hyponatremia in people concomitantly using MDMA and alcohol with the aim to support the development of prevention and harm-reduction strategies, and to increase the clinical safety of MDMA in future therapeutic applications.

## Methods

**Fig. 1:**
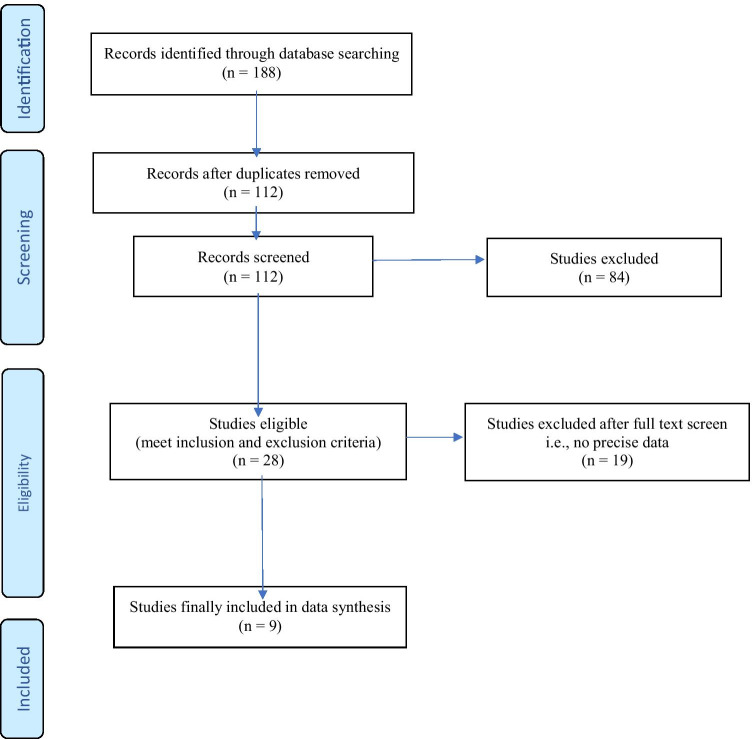
PRISMA flow diagram. Additional eligible reports were retrieved via the checking of reference list of the selected 78 studies, including the reviews

A systematic literature review was performed using the PRISMA-protocol to retrieve studies from Medline (PubMed), EMBASE, and PsycINFO. Relevant studies were collected up to May 23, 2021, including articles published or ahead of print from September 2010 to October 2020. Inclusion criteria were as follows: (1) describes the relationship between the combined use of MDMA and alcohol with either hyperthermia, hyponatremia, or AVP-release and (2) data reported full text in either Dutch, English, German, or French. Exclusion criteria were commentaries and case reports. Two researchers (JvA and MP) were involved in the selection of appropriate studies, which was executed in two rounds. Initially, 188 studies were retrieved of which 112 unique studies remained after removal of duplicates. These 112 studies were further processed; i.e., title and abstract were screened to determine eligibility applying the inclusion and exclusion criteria mentioned above. In a second round, full texts of 28 selected were checked for eligible studies resulting in the final inclusion of nine papers. Figure 1 shows the PRISMA diagram of the identification, screening, and inclusion of the reports. See figure “Supplementary information” for the search string and PRISMA checklist.

Because the content in the nine selected papers contained limited information on the possible mechanisms related to the combined effect of MDMA and alcohol on hyperthermia and hyponatremia, we also searched the reference lists of three reviews about the interaction between MDMA and alcohol (Mohamed et al. 2011; Papaseit et al. 2020; Vercoulen and Hondebrink 2021) and the reference lists of the papers that were retrieved in our systematic search but were not included. These additional papers provided relevant information to answer our research question.

## Results

First, we describe the results of the systematic research on the interaction between MDMA and alcohol on hyperthermia and hyponatremia (“Effect of Concomitant Alcohol Use on MDMA-Induced Hyperthermia and Hyponatremia”). Then we summarize what is generally known about regulation of body temperature and hydration (“Mechanisms Involved in the Increased Risk of MDMA and Concomitant Alcohol Use”). Finally, we describe the literature on the possible mechanisms involved in the combined effects of MDMA and alcohol on hyperthermia (“Regulation of body Temperature”), dehydration (“Effect of MDMA and/or Alcohol on Body Temperature”), and hyponatremia (“Effect of MDMA and/or Alcohol on Hydration”).

### Effect of Concomitant Alcohol Use on MDMA-Induced Hyperthermia and Hyponatremia

Table 2 describes the results of the nine studies finally included (one human study and eight rodent studies). Overall, MDMA induced a modest to strong increase in body temperature, which was attenuated by alcohol under normal ambient temperatures, but not under relatively high ambient temperature conditions. In the human study, MDMA-induced minor hyperthermia (+ 0.4 °C) and this effect tended to be attenuated (*P* = 0.09) by pre-treatment with alcohol (0.6‰ v/v) (Dumont et al. 2010). At ambient temperatures of 21–23 °C, a high dose of ethanol (1.5 mg/kg) attenuated MDMA-induced hyperthermia in rats for several hours (Cassel et al. 2004, 2005; Hamida et al. 2007, 2006, 2008), although in some of these studies, tolerance of the alcohol effect was observed. One rodent study (Cassel et al. 2007) showed that the same dose of MDMA caused a much stronger hyperthermic response at an ambient temperature of 32 °C compared to an ambient temperature of 23 °C and that the concomitant use of alcohol had no attenuating effect at high ambient temperatures. Similarly, pre-treatment with alcohol at a high ambient temperature (30 °C) did not attenuate the hyperthermic effect of MDMA in humans (Izco et al. 2007). Finally, it is of interest that all rodents treated with MDMA (6.6 mg/kg i.p.) at 32 °C died after 120 min, whereas no lethality was observed with the same dose of MDMA at 23 °C (Cassel et al. 2007). In summary, these studies show that alcohol has an attenuating effect MDMA-induced hyperthermia in humans and rodents in rest and under normal ambient temperatures. In contrast, at (relatively) high ambient temperatures and during exercise, MDMA induced serious and often fatal hyperthermia in rodents which were not prevented or attenuated by concomitant use of alcohol (see Table 2; “Effect of MDMA and/or Alcohol on Hydration”). Table 2 also shows that no data were found about the effect of concomitant alcohol use on MDMA-induced hyponatremia.

**Table 2:** Studies describing the effect on temperature by MDMA either given simultaneously with alcohol (EtOH) or following pre-treatment with ethanol (EtOH) in rodents

Nr	Treatment	Observations	References
1	MDMA (5 mg/kg) following 4-day treatment with EtOH (plasma level: 4.5 mg/ml) at 30 °C	Similar hyperthermia (+ 1.5 °C) as controls	Izco et al. (2007)
2	16 healthy subjects; MDMA (100 mg p.o.) while their blood EtOH concentration was 0.6‰ and ambient temperature was 22 °C	EtOH tended (*P* = 0.09) to attenuate the limited MDMA-induced temperature increase (+ 0.4 °C)	Dumont et al. (2010)
3	EtOH (1.5 g/kg) + MDMA (6.6 mg/kg) for 4 days at 23 °C	Partial inhibition of MDMA-induced hyperthermia by EtOH, an effect persisting after day 1	Hamida et al. (2007)
4	EtOH (1.5 g/kg) for 4 days followed by MDMA (6.6 mg/kg) at 23 °C	EtOH attenuated MDMA-induced hyperthermia	Hamida et al. (2006)
5	EtOH (1.5 g/kg) + MDMA (6.6 mg/kg) on four occasions (2, 5, and 2 days apart) at 23 °C	EtOH attenuated MDMA-induced hyperthermia (+ 1 to + 1.5 °C), an effect increasing across treatment days	Hamida et al. (2008)
6	EtOH (1.5 g/kg) + MDMA (10 mg/kg) for 4 days at 23 °C	On day 1, EtOH inhibited the MDMA-induced hyperthermia, but not on the subsequent days when MDMA with or without EtOH induced hyperthermia (+ 1.9 °C)	Cassel et al. (2004)
7	EtOH (1.5 g/kg) and/or 10 mg/kg MDMA for 4 days at 23 °C	EtOH attenuated the MDMA-induced hyperthermia (2.2 °C), but only on day 1	Cassel et al. (2005)
8	EtOH (1.5 g/kg) + MDMA (6.6 mg/kg i.p.) at 32 °C	Both MDMA and EtOH + MDMA-induced severe hyperthermia (+ 2.7 °C and + 2.4 °C, respectively). All rats died after 120 min, whereas no lethality at 23 °C and EtOH reduced the hyperthermia	Cassel et al. (2007)
9	EtOH (20% v/v) in drinking water for 2 h on 4 consecutive days. On day 4: MDMA (20 mg/kg) was tested at 22 °C	MDMA-induced hyperthermia (+ 0.5 °C) in mice pre-exposed to water, but induced hypothermia (− 0.8 °C) in mice pre-exposed to binge ethanol	Ros-Simó et al. (2012)

### Mechanisms Involved in the Increased Risk of MDMA and Concomitant Alcohol Use

**Fig. 2:**
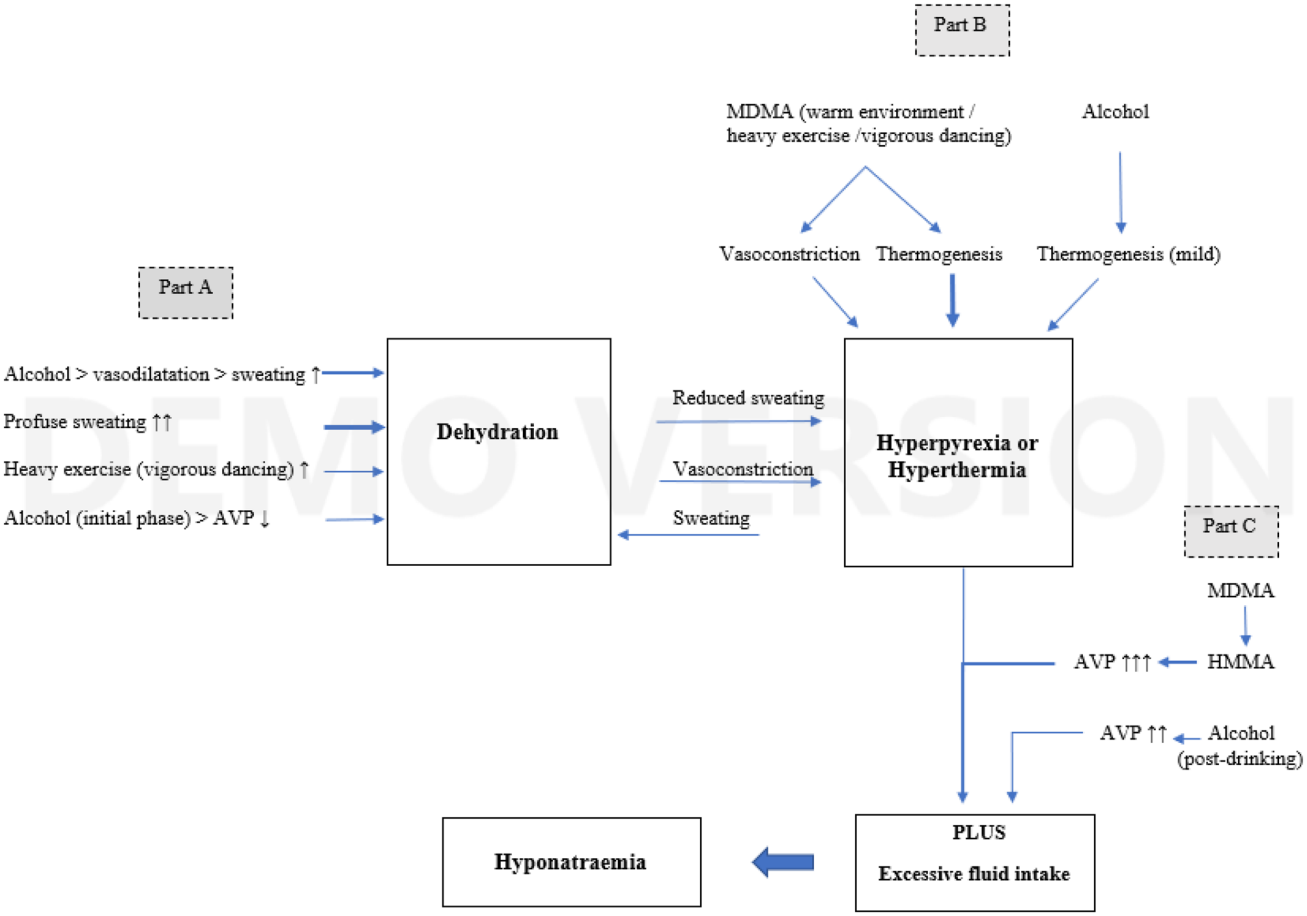
Mechanisms involved in hyperthermia and hyponatremia induced by MDMA and alcohol. Part A: sweating and vigorous dancing results in heavy sweating and dehydration. Dehydration is further increased by increased sweating as a result of vasodilatation and reduced AVP-secretion (arginine vasopressin) by alcohol. Hyperpyrexia results from (a) dehydration-induced reduced sweating and vasoconstriction in dehydrated subjects and, as depicted in Part B, MDMA induced vasoconstriction and thermogenesis (under unfavorable conditions). Part C: HMMA (4-hydroxy-3-methoxy-methamphetamine), the major metabolite of MDMA, is a potent releaser of AVP. In addition to hyperthermia, the increased secretion of AVP, by both alcohol and MDMA, is responsible for excessive fluid intake leading to hyponatremia

Hyperthermia, dehydration, and over-hydration (i.e., hyponatremia) are the main causes of MDMA-related life-threatening events or even death (Kalant 2001). MDMA may induce hyponatremia without symptoms of hyperpyrexia, but hyponatremia can also be elicited secondary to hyperpyrexia. Below we will further elaborate on the propagating and facilitating effects of alcohol on MDMA-induced hyperthermia and hyponatremia which explain the increased risk of the combined use of MDMA and alcohol. The various determinants involved in MDMA-induced hyperthermia and hyponatremia are summarized in Table 3 and some of the mechanisms are depicted in Fig. 2. Figure 2 outlines that although concomitant alcohol use may have an *attenuating effect* of MDMA-induced hyperthermia (previous paragraph), it may — under certain conditions — have *detrimental effects* on both MDMA-induced hyperthermia and hyponatremia.

**Table 3:** Determinants putatively involved in MDMA-induced hyperthermia or hyponatremia

	Determinant	Result	References
1	Dysregulation of the thermoregulatory system (poikilothermia)	Hyperpyrexia at high ambient temperature	Docherty and Green (2010); Rusyniak et al. (2008)
2	Warm ambient temperature and heavy exercise (vigorous dancing)	Increased heat generation in adipose tissue and skeletal muscles	Mills et al. (2003); Rusyniak et al. (2005)
3	Peripheral vasoconstriction	Reduced peripheral blood flow > lower heat dissipation	Gordon et al. (1991); Pedersen and Blessing (2001)
4	Water management in the body	If dehydrated, less water available for sweating. Dehydration via inhibition of AVP-release by diuretics, like alcohol, caffeine (and MDMA)	Camarasa et al. (2006); Vanattou-Saïfoudine et al. (2012)

### Regulation of body Temperature

#### Balance Between Heat Production and Dissipation

Hyperthermia (heat stroke) emerges from an imbalance between the accumulation of heat generated in and the capacity to dissipate heat from the body. Heat is dissipated in humans by peripheral vasodilatation and sweating. Evaporation of 1 ml of sweat from the skin represents the loss of approximately 0.58 kcal (Murray 1996). In a hot environment, the sweat rate can exceed 2500 mL/h (Sawka and Pandolf 1990), which equals a heat dissipation rate of 1450 kcal/h (6000 kJ/h). As such, sweating represents a rapid and huge heat transfer to the environment. In response to massive water loss due to sweating, AVP is secreted from the pituitary gland. It is interesting to note that rodents miss the capacity to sweat.

#### Thermoregulation

Humans are homeothermic animals, and their (core) temperature is relatively stable under changing ambient temperatures. Temperature regulation in man is under control of the thermoregulatory center in the hypothalamus. Hyperthermia typically involves a failure or disruption of this control system. Poikilothermic (poikilos means “manifold”) agents render subjects poikilothermic, whereby they tend to equilibrate their body temperature with the ambient temperature they are in. Under such conditions, a cool environment results in hypothermia and a warm environment in hyperthermia. As explained below, both MDMA and alcohol are poikilothermic agents, i.e., agents that disrupt the thermoregulatory control system.

Only organs of sufficient metabolic capacity, like brown adipose tissue (BAT) and skeletal muscle (SKM), can generate large amounts of heat (thermogenesis). For example, rapid muscle contraction or shivering generates considerable heat in SKM, but such shivering is energetically costly and impractical to sustain for extended periods of time. However, excessive heat production in BAT and SKM (tremors, muscle cramping) causes hyperthermia which in turn increases the risk of organ damage (Rosenberg et al. 1986; Rusyniak and Sprague 2006). BAT is densely innervated by sympathetic neurons, and the binding of noradrenaline (NA) to β_3_-adrenoceptors, predominantly present in adipose tissue (Skeberdis 2004), is most significant for thermogenesis (Cannon and Nedergaard 2004; Dao et al. 2014). NA can also stimulate alpha_1_-adrenoceptors causing vasoconstriction so that heat dissipation via the skin is blocked and body temperature is increased (Mills et al. 2004; Zhao et al. 1997). Due to excessive dancing under the influence of MDMA, SKM generates heat in the muscles on top of that produced by BAT (Liechti 2014). The concomitant increase in heartbeat and blood flow generates even more heat.

MDMA, a potent noradrenaline and serotonin reuptake inhibitor, has shown to promote thermogenesis and to induce hyperthermia. This is presumably mainly caused by increased sympathetic outflow, considering that carvedilol, an antagonist of both *α*_1_ and *β*_3_-adrenoreceptors and the *β*_3_-adrenoreceptor blocker cyano-pindolol, but not nonselective (*β*_1_ + *β*_2_) adrenoceptor antagonists, attenuates the MDMA-induced increase in body temperature (Hysek et al. 2012, 2013; Kiyatkin et al. 2016; Sprague et al. 2005). For instance, the increased in rectal temperature (+ 3.6 °C) by MDMA (40 mg/kg, s.c.) was inhibited by the *α*_1_-adrenergic antagonist prazosin together with the *β*_3_-adrenergic antagonist SR59230A (Bexis and Docherty 2009; Sprague et al. 2004). In addition, MDMA induced peripheral vasoconstriction, limiting heat dissipation (Gordon et al. 1991; Pedersen and Blessing 2001).

The role of serotonin (5-HT) in thermoregulation is more complicated with amongst others the 5-HT_2A_ receptor as a key factor in the development of hyperthermia (Herin et al. 2005). Serotonin, released by the hypothalamus (De Fanti et al. 2000) or direct injection of serotonin into the hypothalamus, activates thermogenesis in BAT (Cannon and Nedergaard 2004) resulting in hyperthermia (Ishiwata et al. 2017). Studies using dopamine antagonists have also indicated the involvement of dopamine in MDMA-induced hyperthermia (Rusyniak et al. 2008). Furthermore, antagonists of both 5-HT_2A_ and D_1_ receptors counteract at the level of the hypothalamus the MDMA-induced hyperthermia (Vanattou-Saïfoudine et al. 2010).

The excessive heat produced (i.e., hyperthermia) disturbs intracellular biochemical signaling throughout the body resulting in devastating medical complications, including multiple organ failure, renal failure, metabolic acidosis, liver failure, intravascular coagulation, and/or rhabdomyolysis (breakdown of damaged skeletal muscle), which may be fatal. Interestingly, most drugs that protect against MDMA-induced neurotoxicity in rodents either cause hypothermia or prevent MDMA-induced hyperthermia (Colado et al. 1999a, b; Colado et al. 1998, 2001; Colado et al. 1999a, b), suggesting that dysregulation of thermoregulation by MDMA is a crucial element of its neurotoxicity.

#### Hydration

MDMA is also a fascinating drug from a hydration physiology perspective, because it can cause the typical symptoms of dehydration, like thirst and dry mouth, whereas it causes hyperhydration on a cellular level. Moreover, dehydration is an important factor in the development of hyperthermia in recreational MDMA users who vigorously dance at relatively high ambient temperatures (Henry 1992; Patel et al. 2005; Tehan et al. 1993). The high sweat rates required to sustain heat loss during exercising/dancing at 25–35 °C (Desruelle et al. 1996) inevitably lead to dehydration, unless sufficient fluid is ingested to supplement the volume of sweat lost. For every 1% of body weight lost (equivalent to around 700 ml of sweat) during exercise, the core body temperature increases by 0.15–0.23 °C (Coris et al. 2004; Sawka 1992). Body fluid losses of around 8% of body mass (equivalent to 5–6 L of sweat) are hazardous and may become fatal if it exceeds this percentage (Grandjean et al. 2003). The magnitude of dehydration accrued after 2 h of exercise at an ambient temperature of 33 °C (no supplementation, and fluid supplementation: 81, 48, 20%) was linearly related with an increase in body temperature (38.4 to 39.1 °C; *r* = 0.98) (Montain and Coyle 1992). Similarly, Graham et al. (2020) showed that rectal temperature increased by 1.1 °C following exercise for 180 min at an ambient temperature of 40 °C, if no fluid was supplemented during exercise. Being dehydrated during exercise at 35 °C resulted in a body temperature of nearly 39 °C, which was considerably higher than in euhydrated subjects (Nadel et al. 1980). Core temperature increased with severity of dehydration, and sweating rate was inversely associated with severity of dehydration (Sawka et al. 1985). Dehydration of approximately 2–3% of body mass routinely occurs in healthy subjects during intermittent high-intensity exercise, especially when the ambient temperature is high (Galloway 1999). Upon dehydration, sweat rate considerably decreases, which in turn may increase body temperature.

MDMA-induced hyperthermia resembles heatstroke (body temperature ≥ 40.0 °C) as observed in athletes running a marathon on warm summer days. Using a simple wrist pedometer, it was shown that an ecstasy user “danced” during one night in the club around 40 km which is virtually the distance of a marathon (WvdB, personal communication). Some 60–80 out of 36,000 marathon athletes were transferred to ED in 2019, because of overheating during a half marathon in Amsterdam on a warm day (24–25 °C) in 2019 (Het Parool 2019). Dehydrated patients may have excessive thirst (Pfennig and Slovis 2012), but the thirst stimulus may not occur until the patient is already 5% dehydrated (AAP 2000), because the response to thirst is desensitized during conditions of physiologic stress. The apparent lack of or delayed awareness of thirst under such conditions therefore facilitates further dehydration and further increases the risk of serious complications (Cole and Sumnall 2003).

Dehydration is commonly accompanied by diminished diuresis (Pfennig and Slovis 2012). AVP serves to limit unnecessary water loss from the body, especially under hot environmental conditions or when much heat is generated by the body. Exposure of humans for 2 h in an environment of 50 °C increased AVP levels from 1.6 to 5.2 μU/ml (Segar and Moore 1968), explaining why men performing work in a hot environment reduced their urine flow, while sweat loss was initially about one liter per hour and water was drunk ad libitum (Weiner 1945).

In summary, dehydration promotes exercise induced hyperpyrexia at high ambient temperatures which coincides with stimulated AVP-secretion.

### Effect of MDMA and/or Alcohol on Body Temperature

#### Human Studies

In humans, some placebo-controlled studies performed at normal ambient temperature showed that MDMA did not increase body temperature, whereas other studies showed a modest elevation of body temperature (Dumont and Verkes 2006; Freedman et al. 2005; Liechti 2014; Liechti et al. 2001; Parrott 2012). Pooled data analyses showed that administration of 125 mg MDMA in a controlled laboratory setting produced an acute and dose-dependent elevation in core body temperature in healthy subjects (Liechti 2014). Importantly, these experiments were performed in subjects at rest and at a mean ambient temperature of 22.7 ± 0.6 °C. This increase in body temperature is rather small (range 0.2–0.8 °C) and did not result in hyperpyrexia (> 40.0 °C). However, a substantial number of the subjects (23%) showed moderate hyperpyrexia (> 38.0 °C), demonstrating that MDMA may induce moderate hyperpyrexia even in the absence of any risk factors and at normal ambient temperature (23 °C) (Liechti 2014). Freedman et al. (2005), studying the physiological effects of MDMA at cold (18 °C) and warm (30 °C) ambient temperatures, showed that MDMA (2 mg/kg) increased in both warm and cold conditions a similar increase in body temperature (0.3–0.6 °C). In general, at low doses and at moderate ambient temperatures, alcohol is known to induce in humans hypothermia (Huttunen et al. 1998) and vasodilatation (Wasielewski and Holloway 2001) with the latter effect responsible for the warm sensation. However, at higher doses and relatively high ambient temperatures, alcohol may cause hyperthermia (Kalant and Lê 1983). Alcohol is therefore referred to as a poikilothermic agent, a substance that causes maladaptation of the body to either cold or warm conditions. The only human study on the concomitant use of MDMA and alcohol showed that — at normal ambient temperature (22 °C) – alcohol tended (*p* = 0.09) to attenuate the limited MDMA-induced increase in body temperature (Dumont et al. 2010).

#### Rodent Studies

Rats repeatedly (3 times) treated with MDMA (4 mg/kg i.p.) showed a larger hyperthermic response (+ 2.6 °C) at 30 °C compared to 19 °C (+ 1.3 °C) (Sanchez et al. 2004). Ambient temperature (11, 24, or 30 °C) significantly affected the magnitude of the hyperthermia induced by 2.5–7.5 mg/kg MDMA with hyperthermia (+ 4 °C at 7.5 mg/kg) only observed at 30 °C (Dafters 1995). MDMA (2.5–7.5 mg/kg) induced hypothermia in rats previously living at 11 °C (Dafters 1994). Other rodent studies employed very high doses which produced hyperthermia and high levels of morbidity and neurotoxicity (Miller and O'Callaghan 1994). For instance, MDMA (30 mg/kg s.c.) increased colonic temperature by 3.2 °C at 30 °C, but there was no change at 20 °C (Gordon et al. 1991); at 10 °C, it even induced hypothermia (− 2.0 °C). MDMA (20 or 40 mg/kg) induced hypothermia in rats at 20 and 22 °C, but hyperthermia at 28 and 30 °C (Malberg and Seiden 1998). MDMA at a moderate non-toxic dose of 9 mg/kg, s.c. induced relatively weak hyperthermia under standard conditions (quiet rest 22–23 °C), but severe hyperthermia in warm environments (29 °C) (Brown and Kiyatkin 2004; Kiyatkin et al. 2014). These observations show that MDMA, like alcohol, is a poikilothermic agent. The most robust hyperthermia was found during social interaction versus no social interaction of rats treated with MDMA (9 mg/kg): + 2.4 °C versus + 0.95 °C. At 29 °C, MDMA increased brain temperatures to fatal values (42.2 °C) versus an increase to 37.7 °C at 22–23 °C: lethality was found for all 6 tested rats under this condition within 6 h post-injection. In rats, the combination of warm temperature (32 °C) and physical activity (treadmill) increased the body temperature more pronounced (+ 4.4 °C) than each factor alone (+ 0.4 and + 1.8 °C, respectively). This synergistic effect was blocked by the 5-HT_2A_ receptor antagonist M100907, but not by the 5-HT_1A_ receptor antagonist WAY100635 (Tao et al. 2015). Pre-treatment with an inhibitor of serotonin synthesis, the serotoninergic antagonist methysergide, or the 5-HT_1A_ antagonist WAY100635 in rats at 30 °C prolonged the MDMA (5 mg/kg i.p.) induced hyperthermia (Saadat et al. 2005). From this result, it was concluded that diminished 5-HT function leads to an inability to control core temperature in rats under influence of MDMA at high ambient temperatures. This is consistent with the theory that detrimental effects on 5-HT nerve endings result in an increase in body temperature, due to dopamine which is no longer sufficiently abated by the 5-HT system (Green et al. 2004a, b). An interesting result in this regard was found in a study into the combined effects of alcohol and MDMA, whereby exposure to alcohol worsened MDMA’s effects on the 5-HT system, reducing 5-HT transporter density (Izco et al. 2007). At relatively high ambient temperature (33–42 °C), alcohol (0.36–0.54 g/kg body weight) was found to modestly increase body temperature (around + 1.0 °C) (Allison and Reger 1992).

In the few rodent studies that were presented before (Table 2), alcohol had an attenuating effect on MDMA-induced hyperthermia in rodents in rest and under normal ambient temperatures. However, the serious and occasional fatal hyperthermia following MDMA administration seen in rodents under (relatively) high ambient temperatures and during exercise were not prevented or attenuated by alcohol.

### Effect of MDMA and/or Alcohol on Hydration

AVP is normally released in response to a drop in blood volume or a rise in plasma osmolality and acts to conserve water via inhibition of diuresis. MDMA stimulates the secretion of AVP, resulting in higher plasma AVP levels accompanied by a small decrease in serum sodium (de la Torre et al. 2004; Fallon et al. 2002; Forsling et al. 2001; Henry et al. 1998). It is, however, unclear whether this is a direct effect of MDMA or an effect secondary to MDMA-induced hyperthermia (Dowling et al. 1987; Forsling et al. 2001; Henry et al. 1998, 1992). HMMA (4-hydroxy-3-methoxymethamphetamine), the major metabolite of MDMA, is about two-fold more potent than MDMA in releasing AVP (Fallon et al. 2002) which may explain why AVP-secretion peaks relatively late: 1–2 h after MDMA ingestion (Forsling et al. 2001). This lag-time may be relevant for the effect of concomitant alcohol use later in an MDMA-session when alcohol is used in the comedown phase. In contrast to MDMA, alcohol shows a biphasic effect on AVP-secretion: alcohol (> 4% w/v) initially inhibits AVP-secretion (Roberts 1963; Rubini et al. 1955; Shirreffs and Maughan 1997), but 30–60 min after alcohol consumption (1.5 g/kg in 6 h; 27 mM), it inhibits plasma AVP secretion i.e., a decrease from 5.7 to 3.3 ng/L within 30 min, but increased to 17 ng/L at 6 h post-alcohol consumption. As a result, alcohol consumption results in a net loss of 900 mL water within 2 h (Eisenhofer and Johnson 1982; Leppäluoto et al. 1992).

### Effect of MDMA and/or Alcohol on Hyponatremia

Synthetic phenethylamines such as cathinones and amphetamines, including met-amphetamine and MDMA, are known for their induction of hyponatremia. The mechanisms involved in this effect of MDMA and possible the preventive measures have been recently reviewed by Faria et al. (2020). Hyponatremia (serum sodium < 130 mEq/L; low serum osmolality) is a potentially fatal complication associated with MDMA use (Faria et al. 2020; Ghatol and Kazory 2012) and results from increased AVP secretion caused by the MDMA metabolite HMMA. The mechanism and facilitating factors of hyponatremia are summarized in Table 4. A dry mouth and the sensation of thirst are common side-effects of MDMA-induced hyperthermia, which easily results in fluid overconsumption (Campbell and Rosner 2008). However, after a certain lag time following MDMA use, MDMA is metabolized to HMMA which stimulates AVP-secretion. Similarly, alcohol stimulates AVP a few hours following consumption, i.e., some hours after the last consumption of alcohol. At that moment, excessive intake of hypotonic liquids to subside thirst and overheating can easily result in hyponatremia and ultimately in cerebral edema. Increased exertion, compounded by MDMA-induced hyperthermia, may be held responsible for significant sweat sodium losses and thus hyponatremia. Hyponatremia symptoms range from headaches and ataxia to seizures, decreased levels of consciousness and, in some instances, death from cerebral herniation, a pattern closely resembling that of severe heatstroke resulting from physical exertion at high ambient temperatures (Ginsberg et al. 1970). It should be noted, however, that in most MDMA-induced hyponatremia cases, hyponatremia was not associated with hyperthermia.

**Table 4:** Mechanisms and additional facilitating factors involved in the pathogenesis of MDMA-associated hyponatremia (Campbell and Rosner 2008; Faria et al. 2020)

Major mechanisms	Facilitating factors
• MDMA-induced secretion of AVP	• False advice to drink a lot of fluids at rave parties
• MDMA-induced thirst	• Variable loss of body water profuse sweating
• Ready availability of fluids	• Consumption of hypotonic fluids (e.g., water)

“Chill-out” rooms at dance events, where the ambient temperature is relatively low and water and sports drinks are readily available, are primarily designed to limit MDMA-induced hyperthermia. To rehydrate following profuse sweating, subjects should drink water or juice to supplement the lost fluid and the consumption of diuresis promoting drinks. High caffeinated energy drinks are, however, discouraged for rehydration, because they may exacerbate MDMA-induced hyperthermia (Camarasa et al. 2006; Vanattou-Saïfoudine et al. 2012).

## Summary and Conclusion

The various determinants putatively involved in MDMA-induced hyperthermia that are currently outlined may explain that — under certain conditions (e.g., high ambient temperature, heavy exercise) — alcohol use poses an increased health risk when concomitantly used with MDMA. Like MDMA, alcohol is a poikilothermic agent, and both substances may generate heat at high ambient temperatures in an uncontrolled manner. This implies that the additional modest increase of thermogenesis by alcohol, as observed at relatively high ambient temperatures, is no longer adequately downregulated by the thermo-control center in the hypothalamus. In addition, several studies have indicated that adverse hyperthermia–related incidents mainly occur following vigorous dancing and sweating at high ambient temperatures. This is partly due to dehydration occurring under such unfavorable conditions, and severely endangering the maintenance of normal body temperature. Because of their euphoric effects, both alcohol and MDMA may promote vigorous dancing at parties. Moreover, MDMA may delay the initiation of sweating, resulting in an above normal increase of body temperature before sweating commenced (Freedman et al. 2005), while alcohol has no effect on sweating delay. Furthermore, alcohol induces peripheral vasodilatation which counteracts the vasoconstrictive effect of MDMA. Individuals using MDMA may become dehydrated and should be drinking water or juice to supplement lost fluids. Drinking alcohol is, however, not helpful, because it results in a further loss of body fluids. Evidently, antidiuretic drugs (including alcohol and caffeine-containing energy drinks) further promote water loss, dehydration, and hyperpyrexia. In this respect, it is of interest that the diuretic drug caffeine failed to alter body temperature, but it potentiated MDMA-induced hyperthermia and significantly increased MDMA lethality (from 22 to 34%) (Camarasa et al. 2006). Alcohol, at least in the initial phase of its consumption, shows a clear antidiuretic effect which promotes hyperpyrexia. In the second phase, alcohol promotes dehydration via (profuse) sweating due to its vasodilatory effect. Dehydration itself may induce vasoconstriction and may as such facilitate MDMA-induced vasoconstriction and hyperpyrexia. In the second phase, alcohol, like MDMA or rather the MDMA-metabolite HMMA, increases AVP production, which may further increase MDMA-induced (severe) hyponatremia.

Both noradrenergic, dopaminergic, and serotoninergic pathways are involved in thermogenesis. MDMA, as a re-uptake inhibitor of these monoamines, may therefore induce profound hyperthermia. The increase in body temperature induced by MDMA is not corrected by thermo-control mechanisms, because MDMA behaves as a poikilothermic substance, implying that such control mechanisms are disrupted. Alcohol, also a poikilothermic substance, may, via its effects on various actors, like dehydration, high ambient temperatures, heavy exercise (vigorous dancing) and vasoconstriction, increase the adverse health effects of MDMA.

It is concluded that recreational users of MDMA should be aware of the risk of concomitant drinking of alcoholic (and caffeinated) beverages for adverse health incidents. This is particularly for the use of this combination under unfavorable environmental conditions, such as high ambient temperatures. Given the low incidence of serous side events seen in recreational users, the controlled clinical use of MDMA seems relatively safe. Still, it is advocated to consider the risk factors currently identified when MDMA is clinically used. This study has some limitations. Firstly, only one of the nine studies about the direct interaction of MDMA and alcohol on hyperthermia, retrieved by the systematic search (cf. Table 2), was performed in humans and the remaining eight studies were performed in rats. This is valuable and relevant, because, in contrast to mice, rats’ physiological and pharmacological pathways resemble more that of humans, implicating that these data are relevant for the human situation. Secondly, the mechanistic studies underlying the conclusions (risks under adverse circumstances) are not based on (a) studies retrieved by a systematic search and (b) only rarely provided direct evidence from observations related to the interaction between the alcohol and MDMA on the main outcomes. As such, the conclusions are based to a large extent on assumptions regarding a combined effect of both agents.

## Supplementary Information

Below is the link to the electronic supplementary material.

Supplementary file1 (DOCX 20 KB)
